# Prognostic and Predictive Value of the American Joint Committee on Cancer Pathological Prognostic Staging System in Nodal Micrometastatic Breast Cancer

**DOI:** 10.3389/fonc.2020.570175

**Published:** 2020-12-18

**Authors:** Jian Shi, Chen-Lu Lian, Feng Chi, Ping Zhou, Jian Lei, Li Hua, Jun Wang, Zhen-Yu He, San-Gang Wu

**Affiliations:** ^1^ Division of Breast Surgery, the University of Hong Kong-Shenzhen Hospital, Shenzhen, China; ^2^ Department of Radiation Oncology, the First Affiliated Hospital of Xiamen University, Xiamen, China; ^3^ Department of Radiation Oncology, Sun Yat-sen University Cancer Center, State Key Laboratory of Oncology in South China, Collaborative Innovation Center of Cancer Medicine, Guangzhou, China; ^4^ Department of Obstetrics and Gynecology, the First Affiliated Hospital of Xiamen University, Xiamen, China

**Keywords:** breast cancer, nodal micrometastasis, AJCC staging, mastectomy, radiotherapy

## Abstract

**Introduction:**

To investigate the prognostic and predictive effect of the American Joint Committee on Cancer (AJCC) 8^th^ edition pathological prognostic staging system in patients with T1-2N1micM0 breast cancer who underwent mastectomy.

**Methods:**

Data from T1-2N1micM0 breast cancer patients who underwent mastectomy from 2010–2014 were obtained from the Surveillance, Epidemiology, and End Results program. The chi-square test, binomial logistics regression, receiver-operating characteristics curve, competing-risk regression model, Cox proportional hazards regression model, and proportional hazard assumption were used for statistical analyses.

**Results:**

We identified 4,729 patients, including 1,062 patients were received postmastectomy radiotherapy (PMRT). Stage change occurred in 88.2% of the patients, of which 84.4% were downstaged and 3.7% were upstaged. Patients with higher pathological prognostic stages were independently predicted to receive PMRT. The 5-year breast cancer-specific survival (BCSS) was 97.5, 93.7, 90.1, 86.0, and 73.5% in disease stages IA, IB, IIA, IIB, and IIIA, respectively, according to the 8^th^ edition criteria (P < 0.001). The AJCC 8^th^ edition demonstrated moderate discriminative ability, and it had a significantly better ability to predict the BCSS than the AJCC 7^th^ edition criteria (P < 0.001). The multivariate prognostic analysis showed that the new pathological prognostic staging was an independent prognostic factor affecting the BCSS. The BCSS worsened with an increase in the stage. The PMRT did not affect the BCSS regardless of the pathological prognostic stage. Similar trends were found using the competing-risks regression model.

**Conclusions:**

The 8^th^ AJCC breast cancer pathological prognostic staging system downstaged 84.4% of patients with T1-2N1micM0 disease and the survival outcome prediction with this staging system was more accurate than the AJCC 7^th^ edition system. Our study does not support using the prognostic stage as a guideline to escalate of PMRT.

## Background

Routine pathological assessment of the prognostic and predictive biological factors of breast cancer, including estrogen receptor (ER), progesterone receptor (PR), human epidermal growth factor receptor 2 (HER2) status, and tumor grade, determine the treatment decision and the response of breast cancers to therapy (1–4). Recognizing this, the American Joint Committee on Cancer (AJCC) 8th edition staging system for breast cancer introduced the clinical and pathological prognostic stages by incorporating biological factors into the traditional anatomic stages (5, 6). Compared with the 7^th^ AJCC anatomic staging system, 8^th^ AJCC pathological prognostic stages incorporating these biological factors provide a more refined prognostication in terms of survival estimates for patients receiving appropriate multidisciplinary treatment (7–10).

With advances in surgical and histopathological techniques of breast cancer, an increased number of patients were diagnosed as having micrometastatic disease (N1mic, ≤2 mm axillary nodal metastasis; 15–20%), which were labeled as node-negative (N0) by routine histological assessment (11–13). However, the prognostic significance for this population remains controversial. Several prior studies have shown comparable disease-free survival (DFS) and/or overall survival (OS) between patients with N1mic and those with N0 disease (14, 15). Other studies have found that N1mic breast cancer does indeed confer a lower survival and that adjuvant therapy should be performed to improve survival outcomes (16–19). The DFS appears to be only slightly lower among N1mic breast cancer patients (20). Also, it remains unknown whether such patients would benefit from postmastectomy radiotherapy (PMRT). In recent years, several studies have attempted to explore the clinical value of PMRT in T1–2 (tumor size ≤5 cm) and N1mic breast cancer, but all studies have yielded negative results (21–25). It is anticipated that the biological factors in breast cancer may inform the decision to carry out PMRT for this population. In light of this, we performed this study to investigate the prognostic effect of the AJCC 8^th^ pathological prognostic staging in T1-2N1micM0 breast cancer patients undergoing mastectomy using a large, population-based cohort. In addition, we also investigated the role of the AJCC 8^th^ pathological prognostic staging on the decision-making of PMRT for this population.

## Methods and Materials

### Patients

Patients were identified from the Surveillance, Epidemiology, and End Results (SEER) database in this study. The SEER database includes cancer incidence and survival information from 18 registries and covers 28% of the United States population (26). We identified female breast cancer patients diagnosed between 2010 and 2014 and met the following criteria: T1-2N1micM0 invasive breast cancer; had undergone mastectomy with and without PMRT; available variables, including age, race/ethnicity, tumor grade, ER, PR, HER2 status, and chemotherapy administration. Patients with metastatic disease at diagnosis, those with no positive pathology diagnosis; unknown radiation status; unknown tumor grade; as well as unknown or borderline ER, PR, and HER2 status were excluded. Because the SEER database contains publicly available data for de-identified patients, Institutional Review Board approval was waived.

The following variables were extracted for analysis: age, race/ethnicity, tumor stage (T1 and T2), tumor grade (grades I, II, and III), hormone receptor status (negative, positive), HER2 status (negative, positive), chemotherapy (no, yes), and PMRT (no, yes). The tumor/node/metastasis (TNM) staging system was based on the anatomic staging according to the AJCC 7^th^ edition staging system, and pathological prognostic staging was determined according to the AJCC 8^th^ edition of the breast staging manual (5, 6). Grade III disease included poorly differentiated and undifferentiated histological grades.

The primary outcomes of this study were breast cancer-specific survival (BCSS) and breast cancer-specific mortality (BSCM). BCSS was estimated from the time of diagnosis of breast cancer to the time of death from breast cancer. Patients who were still alive at the last follow-up or died of other causes were excluded from the analysis. BCSM was defined as the interval from the initial diagnosis of breast cancer to the date of death from breast cancer.

### Statistical Analysis

Descriptive statistics were compared between patients with and without PMRT using the chi-square test. Independent predictive factors that correlated with PMRT were investigated using binomial logistics regression. BCSS curves were generated by the Kaplan-Meier method and compared using the log-rank test. A competing-risk regression model was used to estimate the cumulative incidence of BCSM. The area under the curve (AUC) was estimated using the receiver-operating characteristics (ROC) curve to investigate the discriminatory ability of 7^th^ and 8^th^ AJCC staging criteria to predict the BCSS. Univariate and multivariate analyses were performed using the Cox proportional hazards regression models and competing-risks regression models in the Cox model framework to determine the predictive performance of variables with respect to BCSS and BCSM, respectively. The proportional hazard assumption was tested both graphically and using the Schoenfield residual test. A two-sided P value of < 0.05 was considered statistically significant. All statistical analyses were performed by SPSS software version 22.0 (IBM Corp., Armonk, NY), Stata/SE version 14 (StataCorp, TX, USA), and R version 3.1.1 (https://www.r-project.org/).

## Results

### Patient Characteristics

A total of 4,729 patients were included in this study. Of these patients, 88.0% (n = 4,161), 86.2% (n = 4,077), 85.3% (n = 4,036), 82.2% (n = 3,885), and 77.2% (n = 3,651) were ER-positive, invasive ductal carcinoma, HER2-negative, grade II–III, and PR-positive, respectively. **Table 1** lists the patient characteristics. Moreover, 22.5% (n = 1,062) and 53.1% (n = 2,513) of the patients received PMRT and chemotherapy, respectively. A total of 2,456 (51.9%) and 2,273 (48.1%) patients were categorized into disease stages IB and IIB, respectively, according to the 7^th^ AJCC staging system. A total of 2,571 (54.4%), 1,201 (25.4%), 373 (7.9%), 407 (8.6%), and 177 (3.7%) patients were categorized into disease stages IA, IB, IIA, IIB, and IIIA, respectively, according to the 8^th^ AJCC pathological prognostic staging system.

**Table 1:** Patient baseline characteristics.

Variables	n	Non-PMRT (%)	PMRT (%)	P
Age (y)				
<50	1,390	954(26.0)	436(41.1)	<0.001
≥50	3,339	2713(74.0)	626(58.9)	
Race/ethnicity				
Non-Hispanic White	3,255	2,541(69.3)	714(67.2)	0.545
Non-Hispanic Black	478	362(9.9)	116(10.9)	
Hispanic (All Races)	542	412(11.2)	130(12.2)	
Other	454	352(9.6)	102(9.6)	
Histological subtype				
Infiltrating ductal carcinoma	4,077	3,176(86.6)	901(84.8)	0.166
Infiltrating lobular carcinoma	505	375(10.2)	130(12.2)	
Other	147	116(3.2)	31(2.9)	
Grade				
I	844	701(19.1)	143(13.5)	<0.001
II	2,369	1,875(51.1)	494(46.5)	
III	1,516	1,091(29.8)	425(40.0)	
Tumor stage				
T1	2,456	2,042(55.7)	414(39.0)	<0.001
T2	2,273	1,625(44.3)	648(61.0)	
ER status				
Negative	568	402(11.0)	166(15.6)	<0.001
Positive	4,161	3,265(89.0)	896(84.4)	
PR status				
Negative	1,078	793(21.6)	285(26.8)	<0.001
Positive	3,651	2,874(78.4)	777(73.2)	
HER2 status				
Negative	4,036	3,159(86.1)	877(82.6)	0.001
Positive	693	508(13.9)	185(17.4)	
7^th^ AJCC stages				
IB	2,456	2,042(55.7)	414(39.0)	<0.001
IIB	2,273	1,625(44.3)	648(61.0)	
8^th^ AJCC stages				
IA	2,571	1,460(65.9)	1,111(44.2)	<0.001
IB	1,201	482(21.8)	719(28.6)	
IIA	373	129(5.8)	244(9.7)	
IIB	407	128(5.8)	279(11.1)	
IIIA	177	17(0.8)	160(6.4)	
Chemotherapy				
No	2,216	1,976(53.9)	240(22.6)	<0.001
Yes	2,513	1,691(46.1)	822(77.4)	

AJCC, American Joint Committee on Cancer; ER, estrogen receptor; HER2, human epidermal growth factor receptor 2; PMRT, postmastectomy radiotherapy; PR, progesterone receptor; T, tumor.

### Stage Migration

Among all patients, 88.2% of patients had stage changed, of which 84.4% were downstaged and 3.7% were upstaged. In the 7^th^ AJCC stages, 93.7% of the stage IB patients were downstaged to stage IA according to the 8^th^ edition criteria, and there were no patients upstaged. Among patients classified as disease stage IIB according to the 7^th^ edition classification, 74.3% of patients were downstaged, of which 11.8, 46.1, and 16.4% of patients were downstaged to disease stages IA, IB, and IIA, respectively, according to the 8^th^ AJCC staging criteria. In addition, 177 (7.8%) patients had been upstaged to stage IIIA disease according to the 8^th^ edition criteria.

### Predictive Factors Associated *W*ith Postmastectomy Radiotherapy

Results of the univariate analysis showed that patients with younger age (< 50 years), higher tumor grade (grade III), larger tumor size (T2), ER-negative, PR-negative, and HER2-positive disease were more likely to receive PMRT (all P < 0.05). In addition, the percentage of patients who received PMRT increased with the staging (P < 0.001). We used two binomial logistic regression models to assess the independent predictive factors related to the receipt of PMRT (**Table 2**). In the first model, without including pathological prognostic staging, the results showed that younger age, invasive lobular carcinoma, grade III, T2, and PR-negative were the independent predictive factors associated with the receipt of PMRT. The new pathological prognostic staging was included in the second model, and the results showed that younger age, invasive lobular carcinoma, and higher pathological prognostic stages were independent predictors of PMRT receipt. Using stage IA as the reference, the odds ratios (ORs) for PMRT receipt in stages IB, IIA, IIB, and IIIA was 1.692 (95% confidence interval [CI] 1.433–1.997), 1.966 (95% CI 1.533–2.520), 1.967 (95% CI 1.541–2.509), and 3.727 (95% CI 2.708–5.130), respectively.

**Table 2:** Predictive factors associated with postmastectomy radiotherapy receipt.

Variables	Model 1			Model 2		
	OR	95%CI	P	OR	95%CI	P
Age (y)						
<50	1			1		
≥50	0.487	0.421–0.565	<0.001	0.487	0.421–0.564	<0.001
Race/ethnicity						
Non-Hispanic white	1			1		
Non-Hispanic black	1.048	0.831–1.322	0.690	1.058	0.840–1.333	0.632
Hispanic (All races)	0.956	0.767–1.192	0.690	0.962	0.772–1.200	0.733
Other	0.928	0.728–1.183	0.547	0.954	0.749–1.216	0.703
Histological subtype						
Infiltrating ductal carcinoma	1			1		
Infiltrating lobular carcinoma	1.361	1.088–1.703	0.007	1.385	1.113–1.724	0.004
Other	0.955	0.632–1.442	0.826	0.967	0.640–1.460	0.872
Grade						
I	1			—		
II	1.156	0.937–1.427	0.177	—	—	—
III	1.499	1.191–1.887	0.001	—	—	—
Tumor stage						
T1	1			—		
T2	1.892	1.638–2.186	<0.001	—	—	—
ER status						
Negative	1			—		
Positive	0.883	0.673–1.157	0.366	—	—	—
PR status						
Negative	1			—		
Positive	0.821	0.691–0.975	0.025	—	—	—
HER2 status						
Negative	1			—		
Positive	1.083	0.891–1.317	0.424	—	—	—
8^th^ AJCC stage						
IA	—			1		
IB	—	—	—	1.692	1.433–1.997	<0.001
IIA	—	—	—	1.966	1.533–2.520	<0.001
IIB	—	—	—	1.967	1.541–2.509	<0.001
IIIA	—	—	—	3.727	2.708–5.130	<0.001

AJCC, American Joint Committee on Cancer; CI, confidence interval; ER, estrogen receptor; HER2, human epidermal growth factor receptor 2; OR, odds ratio; PR, progesterone receptor; T, tumor.

### Survival and Prognostic Analysis

**Figure 1:**
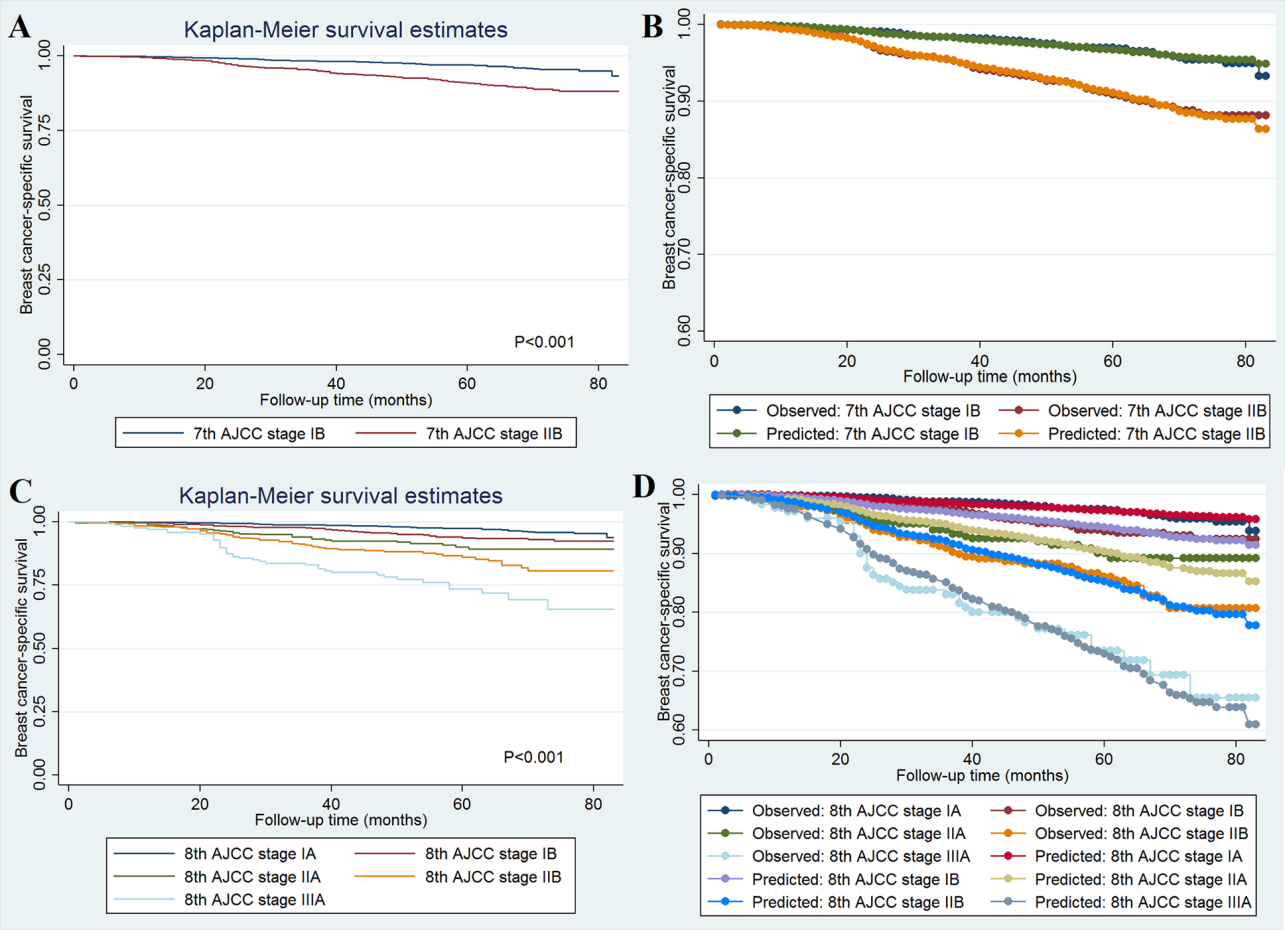
Breast cancer-specific survival curves and the evaluation of the proportional hazards assumption in survival analysis by the AJCC 7th edition **(A, B)** and 8th edition **(C, D)** staging systems.

**Figure 2:**
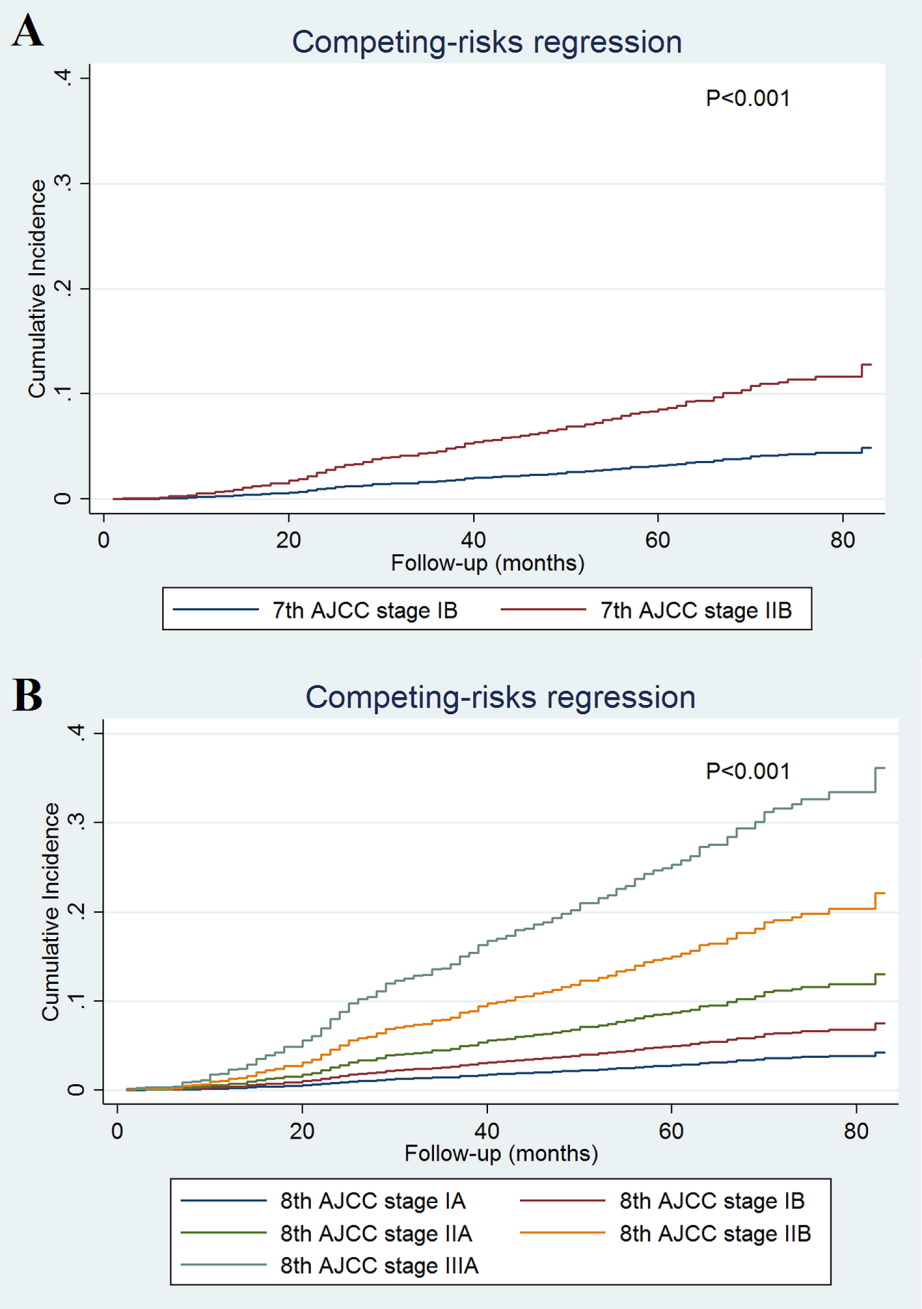
Cumulative incidence of breast cancer-specific mortality by the AJCC 7th **(A)** and 8th **(B)** edition staging systems.

A total of 493 deaths occurred at a median follow-up of 49 months. Of these, 234 (47.5%) and 259 (52.5%) of patients died of breast cancer and other causes, respectively. The 5-year BCSS for stages IB and IIB disease in the 7^th^ AJCC classification system were 97.0 and 90.8%, respectively (P < 0.001; **Figure 1A**). The 5-year BCSS were 97.5, 93.7, 90.1, 86.0, and 73.5% in stage IA, IB, IIA, IIB, and IIIA breast cancer classified according to the 8^th^ edition criteria, respectively (P < 0.001; **Figure 1C**). When staged using the 7^th^ edition AJCC system, the 5-year cumulative incidence of BCSM was 2.9% and 8.8% for stages IB and IIB, respectively (P < 0.001; **Figure 2A**). When staged using the 8^th^ edition criteria, the 5-year cumulative incidence of BCSM was 2.5, 6.0, 9.7, 13.2, and 25.4% in stages IA, IB, IIA, IIB, and IIIA breast cancer, respectively (P < 0.001; **Figure 2B**), which were similar to the results using Kaplan-Meier analysis.

Univariate and multivariate analyses were performed to determine the prognostic performance of variables with respect to BCSS and BCSM, respectively. As more than half of the deaths did not occur from breast cancer, we used two multivariate prognostic models, including the Cox regression model and the competing-risks regression model to assess the independent risk factors influencing patient survival. Univariate analysis showed that the prognostic staging was associated with patient survival (**Table 3**). The results of both multivariate prognostic models also revealed that pathological prognostic staging was an independent prognostic factor of patient survival. As the stage increased, the BCSS decreased (Cox regression model) and the risk of BCSM increased (competing-risks regression model) (**Table 3**). In addition, age was an independent risk factor affecting BCSM. However, PMRT was not found to affect patient survival in either model. Similarly, chemotherapy also did not affect patient survival.

**Table 3:** Univariate and multivariate prognostic analysis using the Cox regression model and competing-risks regression model.

Variables	Cox regression model (unadjusted)		Cox regression model(adjusted)		Competing-risks regression model (unadjusted)		Competing-risks regression model (adjusted)	
	HR (95%CI)	P	HR (95%CI)	P	sdHR (95%CI)	P	sdHR (95%CI)	P
Age (y)								
<50	1		1		1		1	
≥50	1.340 (0.992–1.809)	0.056	1.348 (0.997–1.824)	0.052	1.277 (0.945–1.724)	0.111	1.382 (1.021–1.869)	0.036
Race/ethnicity								
Non-Hispanic white	1		1		1		1	
Non-Hispanic black	1.238 (0.828–1.850)	0.298	1.106 (0.738–1.657)	0.625	1.242 (0.829–1.859)	0.293	1.110 (0.736–1.681)	0.621
Hispanic (All races)	1.349 (0.923–1.973)	0.122	1.164 (0.794–1.706)	0.437	1.372 (0.939–2.006)	0.102	1.191 (0.815–1.740)	0.366
Other	0.779 (0.466–1.302)	0.3400	0.722 (0.431–1.210)	0.216	0.796 (0.477–1.331)	0.385	0.738 (0.439–1.242)	0.252
Histological subtype								
Infiltrating ductal carcinoma	1		1		1		1	
Infiltrating lobular carcinoma	0.714 (0.446–1.142)	0.160	0.754 (0.467–1.218)	0.248	0.717 (0.449–1.147)	0.166	0.777 (0.478–1.262)	0.308
Other	0.996 (0.492–2.019)	0.992	0.902 (0.444–1.834)	0.777	1.010 (0.497–2.051)	0.978	0.923 (0.453–1.881)	0.825
Pathological stage								
IA	1		1		1		1	
IB	2.042 (1.412–2.953)	<0.001	2.051 (1.418–2.966)	<0.001	2.064 (1.426–2.986)	<0.001	2.013 (1.376–2.946)	<0.001
IIA	3.631 (2.337–5.643)	<0.001	3.693 (2.376–5.741)	<0.001	3.688 (2.366–5.748)	<0.001	3.530 (2.242–5.559)	<0.001
IIB	5.721 (3.919–8.351)	<0.001	5.594 (3.830–8.171)	<0.001	5.614 (3.837–8.214)	<0.001	5.222 (3.498–7.795)	<0.001
IIIA	11.280 (7.561–16.828)	<0.001	11.473 (7.688–17.122)	<0.001	11.275 (7.536–16.870)	<0.001	9.933 (6.403–15.408)	<0.001
Chemotherapy								
No	1		1		1		1	
Yes	1.667 (1.270–2.188)	<0.001	1.113 (0.822–1.506)	0.489	1.759 (1.339–2.311)	<0.001	1.196 (0.880–1.627)	0.252
PMRT								
No	1		1		1		1	
Yes	1.388 (1.044–1.845)	0.024	1.096 (0.814–1.476)	0.545	1.420 (1.069–1.888)	0.016	1.104 (0.830–1.469)	0.496

CI, confidence interval; HR, hazard ratio; PMRT, postmastectomy radiotherapy; sdHR, sub-distribution hazard ratio; T, tumor.

The effect of 7^th^ (**Figure 1B**) and 8^th^ (**Figure 1D**) AJCC staging on BCSS met the proportional hazard assumption, which indicated that the constant hazard ratios from the Cox model were reliable. The ROC was assessed using BCSS as the dependent variable, and the 8^th^ edition AJCC staging system demonstrated moderate discriminative ability [AUC = 0.711, standard error (SE) = 0.018, 95% CI 0.698–0.724], which was significantly better than the 7^th^ edition AJCC staging system in predicting the BCSS (AUC = 0.625, SE = 0.015, 95% CI 0.611–0.639; P < 0.001).

### Effect of Postmastectomy Radiotherapy by Pathological Prognostic Stages

**Figure 3:**
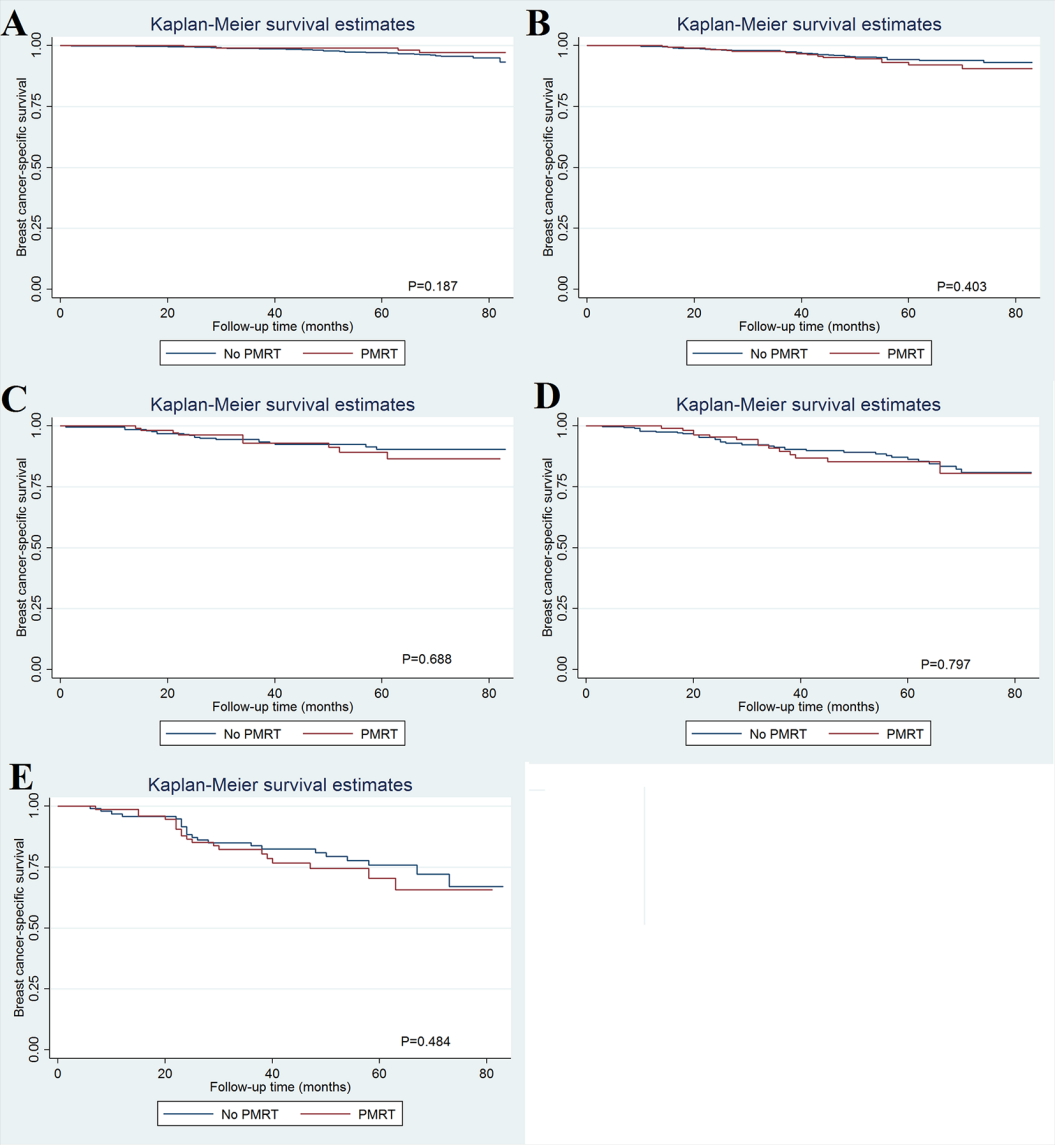
Effect of postmastectomy radiotherapy on breast cancer-specific survival by different pathological prognostic stages (**A**, stage IA; **B**, stage IB; **C**, stage IIA; **D**, stage IIB; **E**, stage IIIA).

**Figure 4:**
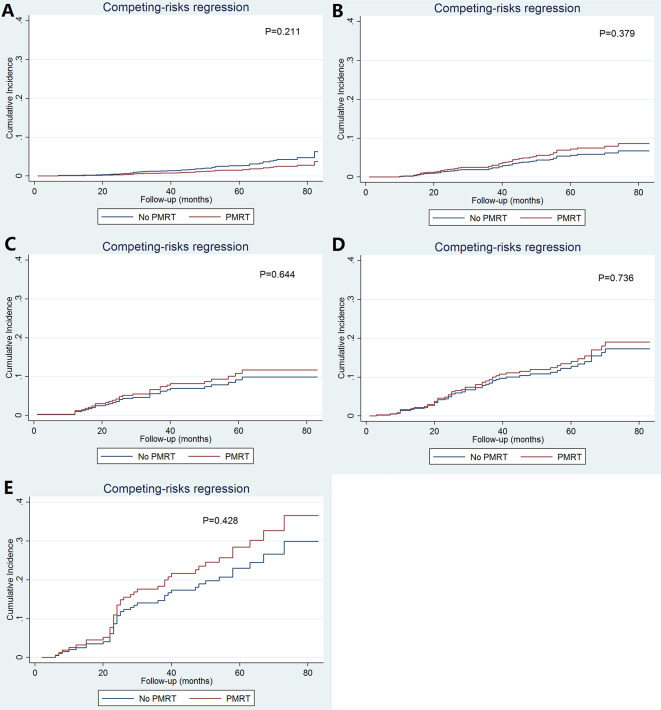
Cumulative incidence of breast cancer-specific mortality with and without postmastectomy radiotherapy according to different pathological prognostic stages (**A**, stage IA; **B**, stage IB; **C**, stage IIA; **D**, stage IIB; **E**, stage IIIA).

As patients with increasing stages were at a higher risk of breast cancer-related death, we further evaluated the value of PMRT in patients with different pathological prognostic stages. Univariate analysis using the Kaplan-Meier method (**Figure 3**) and competing-risk regression model (**Figure 4**) did not find any association between BCSS survival and PMRT in different pathological prognostic stages. The details of univariate Cox regression analysis and competing-risks regression analysis were showed in **Table 4**. After adjusting for age, race/ethnicity, histology, and chemotherapy, PMRT receipt was also not associated with better BCSS or lower BCSM compared to no PMRT, regardless of the pathological prognostic stage (**Table 4**).

**Table 4:** Univariate and multivariate prognostic analysis using the Cox regression model and competing-risks regression model by pathological prognostic stages.

Variables	Cox regression model (unadjusted)		Cox regression model (adjusted)		Competing-risks regression model (unadjusted)		Competing-risks regression model (adjusted)	
	HR (95%CI)	P	HR (95%CI)	P	sdHR (95%CI)	P	sdHR (95%CI)	P
IA								
PMRT Yes vs. No	0.559 (0.240–1.302)	0.178	0.469 (0.199–1.103)	0.083	0.583 (0.251–1.357)	0.211	0.477 (0.208–1.096)	0.081
IB								
PMRT Yes vs. No	1.276 (0.720–2.260)	0.404	1.411 (0.771–2.580)	0.264	1.291 (0.730–2.285)	0.379	1.393 (0.744–2.609)	0.300
IIA								
PMRT Yes vs. No	1.168 (0.547–2.495)	0.689	1.307 (0.597–2.860)	0.504	1.195 (0.562–2.538)	0.644	1.332 (0.656–2.704)	0.427
IIB								
PMRT Yes vs. No	1.085 (0.584–2.015)	0.797	1.242 (0.644–2.394)	0.518	1.111 (0.601–2.055)	0.736	1.222 (0.642–2.326)	0.541
IIIA								
PMRT Yes vs. No	1.245 (0.671–2.309)	0.487	1.265 (0.659–2.429)	0.48	1.279 (0.695–2.354)	0.428	1.279 (0.673–2.429)	0.453

CI, confidence interval; HR, hazard ratio; PMRT, postmastectomy radiotherapy; sdHR, sub-distribution hazard ratio.

## Discussion

The traditional anatomic TNM staging system might not be enough to predict the prognosis and make treatment decisions in breast cancer. The AJCC 8^th^ edition staging system, which includes various biomarkers, could better reflect the prognostic information and selection of therapy for breast cancer (5, 6). In this study, we verified the prognostic effect and predicted the survival benefits of PMRT using the new pathological prognostic system in T1-2N1micM0 breast cancer. Our results indicated that the AJCC 8^th^ edition staging system could refine the prognostic information for this population, but PMRT was not associated with better BCSS regardless of the pathological prognostic stages.

Regarding the 7^th^ AJCC staging system, T1-2N1micM0 was represented by disease stages IB and IIB. We sought to determine the probability of patients staged with the 7^th^ edition criteria to be restaged according to the new pathological prognostic stages. Several population-based and larger cohort studies with stages I–III patients have shown that 36.6–53.2% of patients were restaged from the 7^th^ anatomic stages to the 8^th^ pathological prognostic stages, of which 19.4–29.7% were downstaged and 6.8–31.2% were upstaged (8, 9, 27). In our study, 88.2% of patients were restaged, including 84.4% and 3.7% of patients were assigned to more and less favorable stages (downstaged and upstaged), respectively. Differences in the distributions of biological factors between stage N1mic and other nodal status may be a possible explanation for the higher percentage of patients who were downstaged in our study. In this study, 88.0% of patients were ER-positive, which was similar to the results from Bae *et al.*, who found that there was a significantly higher percentage of ER-positive (87.4% vs.75.9–81.3%) and PR-positive (75.7% vs. 62.4–73.0%) diseases among patients with N1mic breast cancer than patients with N0 and N1a breast cancers (28).

The 8^th^ AJCC pathological prognostic stages were the first time incorporated biological factors, including ER, PR, HER2, and tumor grade into the staging classification system. Several recent studies have confirmed that the new staging system is more reliable than the AJCC 7^th^ edition system for accurately predicting the survival outcome of breast cancer patients (7–10). Similarly, our study also revealed that the 8^th^ AJCC pathological prognostic staging system provided a better distinguish of survival outcomes compared with the 7^th^ AJCC staging system, suggesting that the 8^th^ edition stages also hold true when adjusted by T1-2N1micM0 breast cancer. When comparing the rates of BCSS and BCSM, the application of the AJCC 8^th^ edition staging system resulted in an incremental reduction in BCSS and an increase in BCSM for each stage increase. The superior fit of the AJCC 8^th^ edition staging system makes it a useful tool to discuss survival for this population. Our findings support the concept that biological factors rather than lower nodal burden are the primary driver of survival in N1mic breast cancer.

Breast cancer is predominantly a disease of aging, and increased age has a direct effect on non-breast cancer mortality. In our study, of the 493 death events, 47.5% of the deaths were from breast cancer, while 52.5% were non-cancer mortality or deaths from other cancers, which was similar to the findings reported in previous studies (29, 30). Therefore, in addition to the Cox regression model, we used the competing-risks regression model to avoid overestimation of the risk of BCSM (31), while similar results were found between the Cox regression model and the competing-risks regression model.

There is a paucity of prospective studies to answer the question regarding the effect of PMRT in T1-2N1micM0 breast cancer. In our study, patients with adverse prognostic factors such as younger age, grade III, larger tumor size, and PR-negative status were more like to receive PMRT. Regarding the pathological prognostic stages, patients with advanced stages were more likely to receive PMRT, suggesting that biological factors were also essential indicators to support the decision to administer PMRT. However, whether PMRT had an impact on the survival of T1-2N1micM0 patients remains controversial. The current guidelines of breast cancer recommend strong consideration of PMRT for patients with T1-2N1 (one to three positive axillary nodes) breast cancer after mastectomy, but whether or not N1mic contributes to the positive lymph node count is uncertain (1). Several studies have attempted to answer this question. Mamtani et al. studied 141 N1mic patients who received mastectomy, most of them received appropriate multidisciplinary treatment, including chemotherapy (95%), anti-HER2 targeted therapy (92% of patients with HER2 positive), and endocrine therapy (96% of patients with ER-positive), and they reported that PMRT was not associated with lower locoregional recurrence (LRR) rate (21). Another large cohort study from the MD Anderson Cancer Center (MDACC) found no difference in the LRR rate among N1mic patients with and without PMRT (22). The National Cancer Database included 14019 T1-2N1micM0 patients who underwent mastectomy, and the probability of PMRT receipt (18.5%) and chemotherapy receipt (59.4%) was similar to those reported in our study (23). The results showed that PMRT conferred no benefit to the OS regardless of patient age, hormone receptor status, and tumor grade (23). Moreover, another study by Patel et al. included 5,878 patients from the SEER database, 20% of whom were treated with PMRT. The results showed that PMRT was not associated with better BCSS and OS (24). Finally, in a multicentric cohort study investigated French patients with N0-1mic breast cancer, more than half of them were treated with PMRT. The results indicated that PMRT was not related to improvement in the survival outcomes irrespective of the number of associated recurrence risk factors (25). In our study, 22.5% of patients received PMRT, and PMRT administration did not lead to any significant effect on the BCSS. Thus, none of the abovementioned studies support the significant effects of PMRT in the LRR, BCSS, and OS of N1mic patients.

We further analyzed the differences in patient survival with and without PMRT across different stages as patient survival significantly differed between the two staging systems used. However, although the 5-year BCSM in stages IIB and IIIA reached 13.2 and 25.4%, respectively, PMRT did not improve survival in these stages. The purpose of PMRT was to reduce the LRR and to improve survival (32). The insignificant improvement of survival in different pathological prognostic subgroups may be related to the extremely low LRR rate. The SEER database does not record LRR information. However, several previous studies have investigated the LRR rate in T1-2N1micM0 patients. Mamtani et al. showed that only 3.5% of 141 patients who did not receive PMRT developed LRR (21). The results from the MDACC also showed a 10-year LRR of 3.8% in patients who underwent sentinel lymph node biopsy alone with no PMRT (22). The results from a French multicentric cohort study also showed only 1% of 5-year LRR in patients with N0-1mic breast cancer (25). Similarly, Bazan *et al.* reported low event rates in N1mic patients after mastectomy, and the 6-year LRR and distant metastasis rates were 0 and 5.8%, respectively, and the LRR and distant metastasis rates showed no significant association with systemic therapy (33). However, we should note that the small number of N1mic patients enrolled in the abovementioned studies. Thus, it was difficult to evaluate the risk of LRR based on biological factors. In our previous study, we performed multigene panel testing based on the 21-gene recurrence score and found that PMRT did not improve the survival of patients with T1-2N1micM0, but only ER-positive and HER2-negative patients were included in this study (34). Appropriate identification of patients with excellent or inferior outcomes is key to identifying patients who may be offered de-escalating and escalating treatment strategies, respectively. Therefore, in the future, more studies are needed to explore the impact of new pathological prognostic staging on decision-making of PMRT in this population.

This is the first study to validate the prognostic effect and determine the survival benefit of PMRT in T1-2N1micM0 breast cancer according to the 8^th^ AJCC pathological prognostic staging system. Although we included a large population-based cohort of patients, our study is limited by its retrospective nature and the potential for selection bias. Second, the SEER database does not include complete details on the specifics of the systemic treatments administered. However, the survival trends by the 8^th^ AJCC pathological prognostic staging system observed in our study indicated that most of the patients included in the analysis should also receive appropriate multidisciplinary treatment. Third, the follow-up time in our study was relatively short, which may impact the prognostic and predictive effect of the pathological prognostic stages for this favorable cohort. Furthermore, our findings are not generalizable to the populations of low- and middle-income countries, wherein routine testing of molecular markers and anti-HER2-targeted therapy may not be available. Finally, the SEER database lacks information on locoregional and distant recurrence data, which has a defined correlation with PMRT in this population.

## Conclusion

In conclusion, the 8^th^ AJCC breast cancer pathological prognostic staging system downstaged 84.4% of patients with T1-2N1micM0 disease and could provide more accurate predictions for the survival outcome compared to the AJCC 7^th^ edition staging system. However, our study does not support the use of pathological prognostic staging as a guideline to offer PMRT. Thus, long-term follow-up studies are necessary to further study the role of PMRT in this population.

## Data Availability Statement

Publicly available datasets were analyzed in this study. This data can be found here: www.seer.cancer.gov.

## Author Contributions

JS, C-LL, FC, Z-HY, and S-GW are lead authors who participated in the manuscript drafting, table/figure creation, and manuscript revision. S-GW and Z-HY aided in the data collection. JW, JL, PZ, and LH are senior authors who aided in drafting the manuscript and manuscript revision. Z-YH and S-GW are the corresponding authors who initially developed the concept and drafted and revised the manuscript. All authors contributed to the article and approved the submitted version.

## Funding

This work was partly supported by the National Natural Science Foundation of China (No. 81872459), the Commission Young and Middle-aged Talents Training Project of Fujian Health Commission (No. 2019-ZQNB-25), and the Natural Science Foundation of Guangdong Province (Nos. 2018A030313666 and 2017A030310422).

## Conflict of Interest

The authors declare that the research was conducted in the absence of any commercial or financial relationships that could be construed as a potential conflict of interest.
